# Protein disulfide isomerase A6 promotes the repair of injured nerve through interactions with spastin

**DOI:** 10.3389/fnmol.2022.950586

**Published:** 2022-08-24

**Authors:** Jianxian Luo, Min Xie, Cheng Peng, Yanming Ma, Ke Wang, Gengxiong Lin, Hua Yang, Tianjun Chen, Qiuling Liu, Guowei Zhang, Hongsheng Lin, Zhisheng Ji

**Affiliations:** ^1^Department of Orthopedics, The First Affiliated Hospital, Jinan University, Guangzhou, China; ^2^Department of Orthopedics, Zhuhai Hospital Affiliated with Jinan University (Zhuhai People’s Hospital), Zhuhai, China; ^3^Orthopedics Department I, Zhuhai Hospital of Integrated Traditional Chinese and Western Medicine, Zhuhai, China

**Keywords:** PDIA6, spastin, endoplasmic reticulum homeostasis, protein interaction, nerve repair, spinal cord injury

## Abstract

The maintenance of appropriate endoplasmic reticulum (ER) homeostasis is critical to effective spinal cord injury (SCI) repair. In previous reports, protein disulfide isomerase A6 (PDIA6) demonstrated to serve as a reversible functional modulator of ER stress responses, while spastin can coordinate ER organization through the modulation of the dynamic microtubule network surrounding this organelle. While both PDIA6 and spastin are thus important regulators of the ER, whether they interact with one another for SCI repair still needs to be determined. Here a proteomics analysis identified PDIA6 as being related to SCI repair, and protein interaction mass spectrometry further confirmed the ability of PDIA6 and spastin to interact with one another. Pull-down and co-immunoprecipitation assays were further performed to validate and characterize the interactions between these two proteins. The RNAi-based knockdown of PDIA6 in COS-7 cells inhibited the activity of spastin-dependent microtubule severing. PDIA6 was also found to promote injured neuron repair, while spastin knockdown reversed this reparative activity. Together, these results thus confirm that PDIA6 and spastin function together as critical mediators of nerve repair, highlighting their potential value as validated targets for efforts to promote SCI repair.

## Introduction

Spinal cord injuries (SCIs) result from serious spinal cord damage, adversely impacting the quality of life of affected patients (Venkatesh et al., 2019). SCI repair processes are closely tied to the maintenance of endoplasmic reticulum (ER) homeostasis (Ohri et al., 2013), as the death of many neurons following SCI is not a direct result of the initial injury but is instead secondary to ER stress and other adverse inflammation and damage-related stimuli (Bisicchia et al., 2022). Indeed, prior studies have documented the impact of ER stress on neurons following SCI (Ohri et al., 2013; Liu et al., 2015), with the inhibition of such ER stress being sufficient to protect against SCI-induced neuronal apoptosis, thereby promoting SCI repair (Bi et al., 2020). The mechanisms underlying ER stress-induced neuronal damage following SCI, however, remain to be fully clarified.

Protein disulfide isomerase 6 (PDIA6, likewise called P5) is a key modulator of ER function (Jessop et al., 2009), contributing to ER stress-driven unfolded protein response (UPR) (Matsusaki et al., 2020). The primary proteins that mediate UPR-associated signal transduction are Inositol-requiring enzyme 1 (IRE1), Protein kinase R (PKR)-like endoplasmic reticulum kinase (PERK), and Activating Transcription Factor 6 (ATF6) (Walter and Ron, 2011). PDIA6 cleaves the disulfide bond within oligomeric IRE1, thereby promoting its inactivation, resulting in downstream IRE1 signal pathway activation (Matsusaki et al., 2020). Through this activity, PDIA6 can limit the activation of the UPR pathway under normal physiological conditions (Eletto et al., 2014). Prior work suggests that PDIA6 plays a role in several neurodegenerative disorders such as Alzheimer’s disease (AD) and Huntington’s disease (HD) (Bai et al., 2015; Montibeller and de Belleroche, 2018).

Spastin is a protein that can sever microtubules, thereby regulating their dynamics and promoting the growth and development of neurons (Ji et al., 2018, 2020). Notably, microtubule dynamics are critical to injured neuron regeneration (Stone et al., 2012), and spastin upregulation has previously been linked to axonal regeneration in damaged neurons (Lai et al., 2020). Spastin gene mutations are also thought to be one of the primary causes of a series of heterogeneous neurodegenerative disorders known as hereditary spastic paraplegia (HSP) (Zhu et al., 2019). These spastin gene mutations can contribute to HSP-related symptoms through both the alteration of ER shape and lipid droplet (LD) dispersion, as both of these phenotypes are microtubule-dependent (Arribat et al., 2020). The ability of spastin to regulate ER morphology through the modulation of microtubule dynamics is also associated with its ability to interact with protrudin (Chang et al., 2013; Vajente et al., 2019).

As these prior studies indicate, both spastin and PDIA6 are important regulators of ER homeostasis, with such homeostasis being essential to functional recovery following SCI (Ji Z. et al., 2021). The specific roles that PDIA6 and spastin play in the context of SCI repair, however, have yet to be clarified. This investigative research was thus developed to study the interactions among these two proteins and their mechanistic functions during the process of repairing injured nerves.

## Materials and methods

### Animals

Clear of certain microorganisms, female Sprague-Dawley (SD) rats (10-weeks-old, 180–230 g; or 1-day old) were acquired from Experimental Animal Center of Sun Yat-sen University. The Research on Animals: (ARRIVE) guidelines were utilized for reporting of *in vivo* experiments to design all animal studies. Separately kept rats were placed in a 25°C ± 3°C environment, accessing water and food at liberty. The studies involving animals were reviewed and approved by Jinan University of ethics committee.

### SCI model establishment

Female rats (10-weeks-old; *n* = 36) were randomized into normal control and SCI model groups, with SCI model rats being further separated into light, moderate, and severe injury subgroups (*n* = 9/group). SCI modeling was achieved *via* injury to the T10 spinal segment as reported previously by Wu et al. (2019). Rats were initially put under anesthesia for a short time *via* intraperitoneal sodium pentobarbital administration (30 mg/kg, Sinopharm Chemical Reagent Co., Ltd., Beijing, China. The T9-11 spinal cord was then exposed through a 2.5 cm longitudinal dorsal incision, with the entirety of the T10 lamina then being removed to expose a ∼2.5 mm × 3 mm spinal region. T10 facets were then fixed using a U-shaped rat stabilizer (University of Louisville) loaded onto the stage of the Louisville Injury System Instrument. Spinal cord height was then adjusted under the impactor using laser guidance, with the depth of impact being adjusted to 0.6, 1.0, or 1.8 mm to simulate light, moderate, or extreme damage. The selected impact level was maintained for 0.5 s, with the impact tip being under the control of a nitrogen tank set to 18 psi (124 kPa). Following injury induction, the stabilizer was separated from the stage, and rats were removed therefrom. The injured spinal segment was then examined, with any bleeding being addressed as appropriate. Then, 3-0 silk sutures were used to close the overlying muscle and skin. Successful SCI model establishment was confirmed based on the observation of peri-wound edema, spinal cord ischemia, delayed paralysis, tail wag reflex, and body and leg swing. Sham-operated control rats underwent total T10 laminectomy but were not subjected to SCI modeling. After surgery, rats were administered gentamicin 2,000 U/d (Chongqing Xianfeng Animal Pharmaceutical Co., Ltd.), and manual pressure was applied to the bladder every 8 h to aid urination until the recovery of spontaneous urination.

### Liquid chromatography-mass spectrometry

For glutathione S-transferase (GST)-spastin pull-down assays, a liquid chromatography-mass spectrometry (LC-MS) approach was employed to detect precipitated proteins. Briefly, proteins from samples of T10-centered spinal cord tissue were resolved using NuPAGE 4–12% gels (Life Technologies), visualized with a Colloidal Blue Staining Kit (Life Technologies), and target protein bands were then excised, digested using trypsin, and the resultant peptides were assessed *via* nanoflow reversed-phase liquid chromatography-tandem MS utilizing an HPLC Ultimate 5600 system (AB SCIEX, CA, United States) with a linear ion trap (ThermoElectron, MA, United States) in data-dependent acquisition mode.

### Pathological analyses

At 72 h post-SCI, rats were euthanized using sodium pentobarbital (30 mg/kg, i.p.), and samples T10 spinal cord tissue were excised for immunofluorescent, immunohistochemical (IHC), and hematoxylin-eosin staining (Wang et al., 2016; Fan et al., 2020; Bai et al., 2021). After isolation, sections were stained using a streptavidin-biotin complex kit. Briefly, endogenous peroxidase activity was initially quenched *via* incubation for 10 min with 3% H_2_O_2_ at 37°C, after which sections were washed thrice with phosphate buffer, blocked for 10 min with normal goat serum at 37°C for 10 min, and probed overnight using appropriate primary antibodies (rabbit polyclonal anti-PDIA6, 1:500, Cat# GB11913, Servicebio, Wuhan, China; mouse monoclonal anti-beta III Tubulin, 1:1,000, Cat# GB12139, Servicebio, Wuhan, China; goat polyclonal anti-Mouse IgG, Alexa Fluor 555, 1:1,000, Cat# ab150118, Abcam; Goat polyclonal anti-rabbit, IgG, Alexa Fluor488 1:1,000, Cat# ab150077, Abcam) at 4°C. Samples were then incubated for 1 h with HRP-conjugated anti-rabbit IgG (1:200; Cat# KIT-5004; Fuzhou Maixin, Fuzhou, China) at 37°C. Diaminobiotamine in PBS was then used to stain sections for 30 s, followed by mounting onto glass slides, ethanol gradient-mediated dehydration, xylene treatment, and slides were then imaged *via* microscopy (Olympus, Tokyo, Japan), with Image-Pro Plus 6.0 (Media Cybernetics, GA, United States) being used to quantify staining data.

### siRNA and plasmid preparation

The PDIA6 cDNA sequence (NM_001004442.1) was cloned into the pGEX-5x-3 (Amersham Pharmacia Biotech, NJ, United States) and pCMV-Tag2 (Stratagene, CA, United States) vectors. Three siRNA() constructs specific for PDIA6 were prepared and synthesized by Guangzhou IGE Biotechnology (Guangzhou, China). GFP-spastin and GST-spastin constructs were additionally prepared. All constructs were validated *via* DNA sequencing. A validated spastin-specific siRNA and corresponding scrambled control siRNA (siRNA NC) constructs were obtained from Shanghai GenePharma (Shanghai, China).

### GST pull-down assays

GST pull-down assays were performed as in prior reports (Ji et al., 2018). Initially, whole samples of spinal cord tissue were disrupted, lysed, and combined with rinsed GST-agarose beads (Invitrogen, CA, United States) at 4°C for 1 h. For 10 min, samples were centrifuged at 1,000 × *g* at 4°C, and supernatants were collected. These steps were repeated one additional time, after which ∼400 μg from each sample was incubated overnight with 200 μL of protein-conjugated beads at 4°C. Samples were then spun for 5 min at 1,000 × *g* at 4°C, with unbound protein then being removed by washing pellets using 1 mL of wash buffer. Samples were then spun again for 1 min at 1,000 × *g* at 4°C, after which precipitate proteins were analyzed *via* Western immunoblotting and MS.

### Neuronal culture, transfection, and injury

After 1-day-old SD rats were euthanized with sodium pentobarbital (30 mg/kg, i.p.), brains were harvested, and tissue samples from the hippocampal region were isolated, after which hippocampal neurons were collected following treatment with 0.125% trypsin (Cat# 25200-072; Gibco, MD, United States). These neurons were seeded on poly-D-lysine (Cat# P6407; Gibco)-coated slides (1 × 10^4^ cells/cm^2^), after which they were added to Neurobasal-A medium (Cat# 17504044; Gibco) supplemented with 2% B27 (Cat# 17504044; Gibco). Following a 48 h incubation, constructs were inserted into these cells *via* calcium phosphate transfection. Briefly, for neurons in 24-well plates, 100 pmoL of siRNA was combined with 37 μL of 2 M CaCl_2_ in sterile deionized water (final volume: 300 μL) and combined with 300 μL of 2 × HEPES-buffered saline. Then, 30 μL of this solution was added to each well in a dropwise manner, followed by incubation for 25 min. After 24 h, neurons were fixed for morphological analyses. A model of neuronal damage was established *via* glutamate treatment. Following culture for 48 h, neuronal culture media was replaced with L-glutamate (120 μmol/L), followed by an additional 12 h incubation at 37°C.

### Morphological analyses

A Carl Zeiss LSM 700 confocal microscope (Zeiss, Jena, Germany) with a 63 × oil objective and 1,024 × 1,024 pixel resolution was used to analyze neuronal morphology. Image-Pro Plus (Media Controbernetics, MD, United States) was employed to analyze neuron lengths and branches. Those neuronal protrusions with a length less than twice their diameter were not included in these analyses.

### Statistical analysis

SPSS v22.0 (IBM Corp., NY, United States) was employed for the entire statistical analyses. Data are given as means ± standard error of the mean (SEM), and were compared *via* One-way ANOVA with *post hoc* LSD, with *P* < 0.05 considered statistically significant.

## Results

### PDIA6 is associated with SCI

**FIGURE 1:**
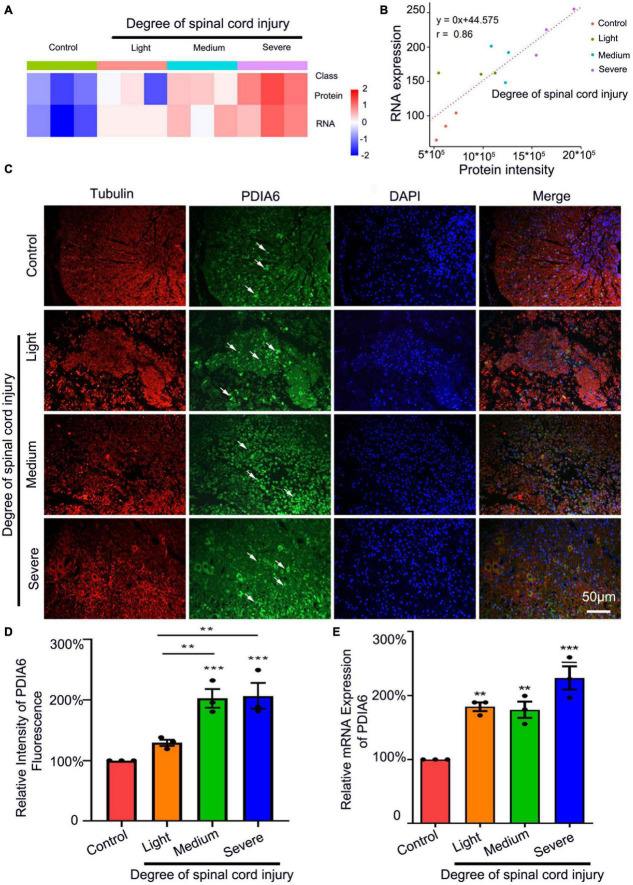
PDIA6 is associated with spinal cord injury. **(A)** Transcriptomic sequencing and LC-MS were used to analyze PDIA6 mRNA and protein expression (*n* = 3/group). **(B)** Correlations between changes in PDIA6 RNA and protein intensity (*n* = 3/group). **(C)** Immunofluorescent staining was used to detect tubulin (red, Alexa Fluor 555), PDIA6 (green, Alexa Fluor 488), and DAPI (blue, Alexa Fluor 405), revealing an increase in PDIA6 expression with greater SCI severity. Scale bar: 50 μm. **(D)** Immunofluorescent PDIA6 staining intensity was quantified, using β3 Tubulin as a control (*n* = 3/group). **(E)** Spinal cord samples from rats subjected to varying levels of SCI severity were collected to assess PDIA6 expression *via* qPCR (*n* = 3 /group). *P < 0.05, **P < 0.01, ***P < 0.01.

Initially, a rat SCI model was established. At 72 h post-injury, transcriptomic sequencing and LC-MS analyses were performed, revealing the upregulation of PDIA6 in a manner correlated with the severity of SCI (Figure 1A and Supplementary Table 1). LC-MS analyses additionally confirmed a positive correlation between PDIA6 mRNA and protein expression in spinal cord tissue samples from model rats subjected to varying levels of SCI severity (Figure 1B and Supplementary Table 1). Immunofluorescent staining of spinal cord sections from these rats similarly revealed higher levels of PDIA6 expression in rats in the moderate and severe SCI groups relative to control animals (Figures 1C,D). Consistently, PDIA6 mRNA levels were elevated in rats with different levels of SCI severity relative to control rats, as demonstrated by qPCR (Figure 1E). These data thus strongly suggest a potential relationship between PDIA6 and the pathogenesis of SCI.

### PDIA6 and spastin interact with one another

**FIGURE 2:**
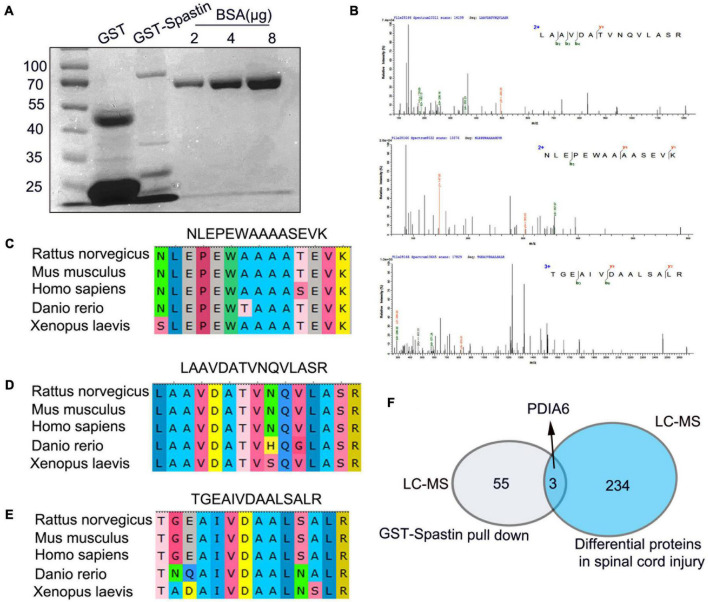
Peptides derived from PDIA6 interact with spastin. **(A)** GST and GST-Spastin protein purification, with BSA serving as a loading control. **(B)** Peptides capable of interacting with GST-spastin were analyzed *via* LC-MS, leading to the identification of three PDIA6-derived peptides (LAAVDATVNQVLASR, NLEPEWAAAASEVK, and TGEAIVDAALSALR). **(C–E)** Significant homology was observed among species when assessing the sequences of the three identified PDIA6-derived peptides. **(F)** PDIA6 is both differentially expressed following SCI and has the potential to interact with spastin.

**FIGURE 3:**
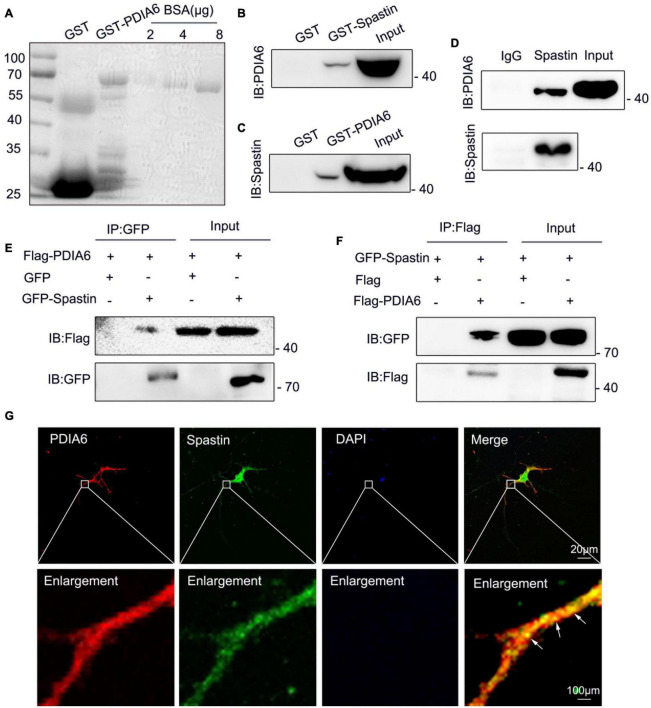
PDIA6 and spastin physically interact with one another. **(A)** GST and GST-PDIA6 protein purification, with BSA serving as a loading control. **(B,C)** Following GST, GST-Spastin, and GST-PDIA6 pulldown of enriched rat brain lysates, PDIA6 and spastin were detected *via* Western immunoblotting. **(D)** Immunoprecipitation assays revealed the ability of PDIA6 and spastin to interact with one another, as proteins that co-eluted with anti-spastin could be detected by both anti-spastin and anti-PDIA6 staining. **(E,F)** COS-7 cells were co-transfected with GFP/Flag-PDIA6, GFP-Spastin/Flag-PDIA6, Flag/GFP-Spastin, or Flag-PDIA6/GFP-Spastin, after which GFP- and Flag-specific antibodies were used for immunoprecipitation. **(G)** Endogenous proteins within hippocampal neurons were detected using anti-PDIA6 (red) and anti-spastin (green). Co-localization between spastin and PDIA6 (yellow) is marked with arrows. Scale bars: 20 μm and 100 μm.

Next, the ability of PDIA6 and spastin to interact with one another was assessed. Following the purification of GST-Spastin and GST-PDIA6 (Figures 2A, 3A), GST-Spastin was utilized to pull down proteins from spinal cord lysates, with precipitated proteins then being analyzed *via* LC-MS. In total, 3 PDIA6 peptides (LAAVDATVNQVLASR, NLEPEWAAAASEVK, and TGEAIVDAALSALR) were found to interact with GST-spastin (Figure 2B), thus validating the capability of spastin and PDIA6 to interact with one another. Sequence homology for these three peptides was further compared among five species (*Rattus norvegicus, Mus musculus, Homo sapiens, Danio rerio*, and *Xenopus laevis*), revealing a high degree of homology consistent with evolutionary sequence conservation (Figures 2C–E). MS analyses further revealed a close relationship between PDIA6 and SCI, suggesting that it may interact with spastin (Figure 2F).

To determine whether PDIA6 and spastin can physically interact with one another, GST-Spastin and GST-PDIA6 were next combined with rat brain lysates to perform pull-down assays, which revealed interactions between these two GST-tagged proteins and PDIA6 and spastin, respectively (Figures 3B,C). Coimmunoprecipitation (Co-IP) assays using these rat brain lysates similarly confirmed the interaction between spastin and PDIA6 (Figure 3D). To further validate this interaction, COS-7 cells were co-transfected using GFP/Flag-PDIA6, GFP-Spastin/Flag-PDIA6, Flag/GFP-Spastin, or Flag-PDIA6/GFP-Spastin, after which GFP- and Flag-specific antibodies were used for pull-down assays. Western immunoblotting revealed an interaction between Flag-PDIA6 and GFP-Spastin (Figures 3E,F). Immunofluorescent staining similarly indicated that PDIA6 and spastin co-localize with one another in neurons (Figure 3G). Together, these data suggested that spastin and PDIA6 can interact both *in vitro* and *in vivo.*

### PDIA6 and spastin interact to control microtubule dynamics

**FIGURE 4:**
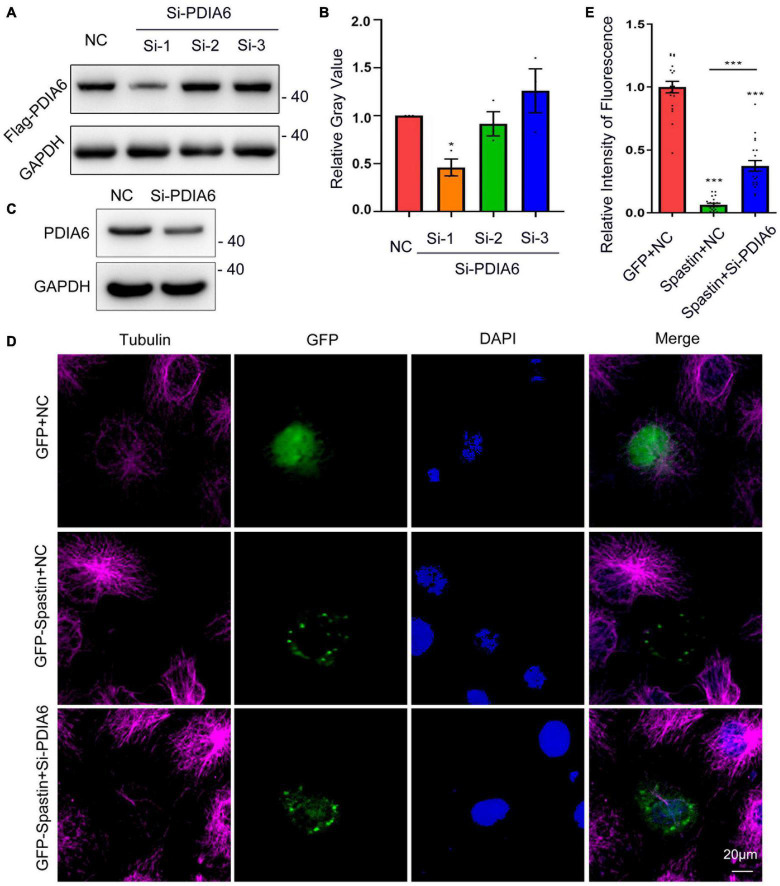
PDIA6 and spastin cooperate to control intracellular microtubule dynamics. **(A)** The efficiency of siRNA-mediated PDIA6 knockdown was examined in COS-7 cells that were co-transfected with three different siRNAs (Si-1, Si-2, or Si-3) and the Flag-PDIA6 plasmid. Anti-Flag was then used for Western immunoblotting, with GAPDH serving as a loading control. **(B)** Relative PDIA6 expression. **(C)** COS-7 cells were transfected using PDIA6 siRNA-1, after which endogenous PDIA6 expression was analyzed. **(D)** Microtubules were detected in COS-7 cells following spastin and Si-PDIA6 transfection; GFP (green), tubulin (Pink), DAPI (blue). Spastin severed microtubules within these cells, whereas the knockdown of PDIA6 disrupted this spastin-mediated severing of microtubules. **(E)** Relative to control cells, those in which PDIA6 was knocked down exhibited significantly reduced spastin fluorescent intensity (*n* = 20/group). *P < 0.05, **P < 0.01, ***P < 0.01.

To additionally examine the effects of PDIA6 on spastin functionality, PDIA6-associated changes in microtubule dynamics were next examined. Three different siRNA constructs were tested for their ability to knock down PDIA6 in COS-7 cells *via* Western blotting (Figures 4A,C), yielding a ∼54% knockdown efficiency (Figure 4B). The effects of PDIA6 on the activity of spastin-dependent microtubule severing were assessed by transfecting COS-7 cells with GFP/NC, GFP-Spastin/NC, or GFP-Spastin/Si-PDIA6 for 24 h (Figure 4D). Relative to control cells or cells overexpressing spastin, the fluorescence intensity of the spastin group reduced considerably. Following si-PDIA6 co-transfection, this fluorescence intensity increased significantly relative to that observed in the spastin group (Figure 4E). These data suggest that spastin is capable of severing microtubules within COS-7 cells, while PDIA6 knockdown was sufficient to inhibit this spastin-mediated microtubule severing activity. The activity of spastin-dependent microtubule severing is thus PDIA6-dependent.

### Interactions between PDIA6 and spastin promote neuronal repair

**FIGURE 5:**
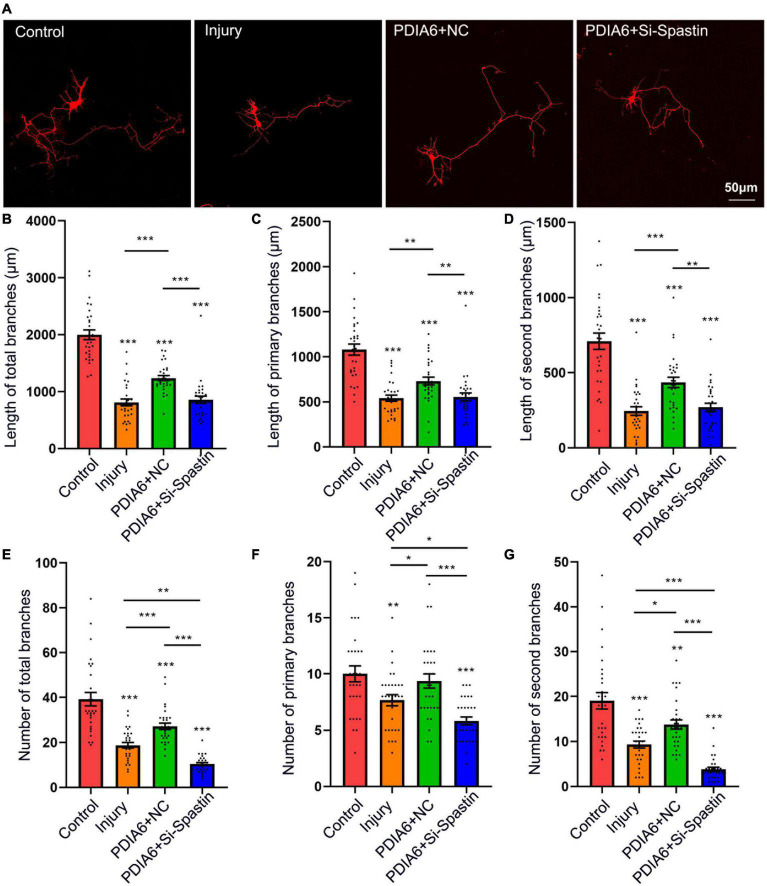
PDIA6 interactions with spastin promote injured neuron repair. **(A)** Following culture *in vitro* for 3 days, glutamate (120 μM) was used to injure hippocampal neurons. At 12 h post-injury, neurons were co-transfected for 24 h with Flag/NC, PDIA6/NC, or PDIA6/Si-Spastin. Scale bar: 50 μm. **(B–D)** Total **(B)**, primary **(C)**, and secondary **(D)** branch lengths were measured, indicating that PDIA6 was able to promote the repair of injured neuron branches, while the silencing of spastin suppressed such PDIA6-mediated repair (*n* = 30/group). **(E–G)** Total **(E)**, primary **(F)**, and secondary **(G)** branch numbers were quantified, indicating that PDIA6 promoted branch formation on injured neurons, whereas the knockdown of spastin inhibited this PDIA6-mediated repair (*n* = 30/group). *P < 0.05, **P < 0.01, ***P < 0.01.

Lastly, the relationship between PDIA6-spastin interactions and neuronal repair was analyzed. To establish a neuronal injury model system, hippocampal neurons were cultured for 72 h *in vitro*, followed by being damaged by exposure to glutamate (120 μM). These neurons were co-transfected with Flag/NC, PDIA6/NC, or PDIA6/Si-Spastin (Figure 5A), with the selected Si-Spastin construct previously having been demonstrated to achieve ∼92% knockdown efficiency in an analysis of the role of spastin as a regulator of neurite outgrowth (Ji et al., 2018). After 24 h of co-transfection, neurites were harvested, and the total length and number of branching neurites were quantified. Cells transfected with PDIA6/NC exhibited a significant increase in the total length of neuronal branches related to the injury and PDIA6/Si-Spastin groups (Figure 5B). Similar results were also observed with respect to primary and secondary neuronal branch lengths (Figures 5C,D). Total numbers of neuronal branches in the PDIA6/NC group were also significantly elevated relative to the injury and PDIA/Si-Spastin in groups (Figure 5E), with similar results being observed with respect to the numbers of primary and secondary branches (Figures 5F,G).

**FIGURE 6:**
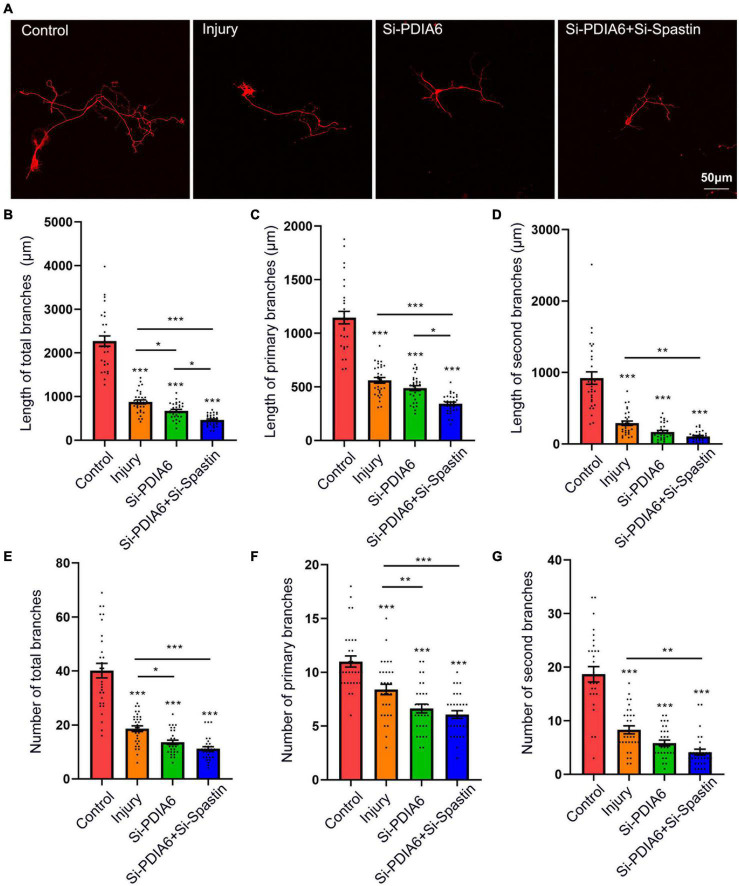
The knockdown of PDIA6 and spastin inhibits injured neuron repair. **(A)** Following culture *in vitro* for 3 days, glutamate (120 μM) was used to injure hippocampal neurons. At 12 h post-injury, neurons were co-transfected for 24 h with Flag/NC, Si-PDIA6, or Si-PDIA6/Si-Spastin. Scale bar: 50 μm. **(B–D)** Total **(B)**, primary **(C)**, and secondary **(D)** branch lengths were measured, indicating that PDIA6 knockdown suppressed branch repair in these injured neurons, while the knockdown of both PDIA6 and spastin suppressed injured neuronal repair (*n* = 30/group). **(E–G)** Total **(E)**, primary **(F)**, and secondary **(G)** branch numbers, indicating that PDIA6 knockdown suppressed branch formation by injured neurons, and that PIDA6 and spastin knockdown similarly inhibited branch formation (*n* = 30/group). *P < 0.05, **P < 0.01, ***P < 0.01.

To further examine the interaction of PDIA6 and spastin in the context of neuronal repair, neurons were co-transfected with the Flag/NC, Si-PDIA6/NC, or Si-PDIA6/Si-Spastin constructs (Figure 6A). Total neuronal branch length was significantly shorter for cells transfected with Si-PDIA6/NC and or-PDIA6/Si-Spastin relative to the injury control group, with a great reduction in branch length in the Si-PDIA6/Si-Spastin group relative to the Si-PDIA6/NC (Figure 6B). Primary neuronal branch length values were additionally shorter in Si-PDIA6/NC and Si-PDIA6/Si-Spastin groups relative to the injury control group (Figure 6C), and secondary neuronal branch lengths were similarly shorter in Si-PDIA6/Si-Spastin group relative to the injury group (Figure 6D). Total branch numbers in Si-PDIA6/NC and Si-PDIA6/Si-Spastin groups were significantly reduced relative to the injury group (Figure 6E), with similar results being observed for the number of primary neuronal branches (Figure 6F). The number of secondary neuronal branches in the Si-PDIA6/Si-Spastin group was also less than that in the injury group (Figure 6G). Together, these data thus confirmed the ability of PDIA6 to promote injured neuron branch repair through interactions with spastin.

## Discussion

Here, PDIA6 was found to play a functional role in the context of SCI, interacting with spastin to promote functional recovery following neuronal injury. Specifically, LC-MS and bioinformatics analyses were initially used to explore proteins that were differentially expressed between healthy and damaged spinal cord tissues, revealing a link between SCI and the expression of PDIA6. PDIA6 was subsequently found to directly physically interact with spastin *in vitro* and *in vivo*, promoting recovery following SCI through the enhancement of neurite outgrowth. Together, these data suggest that PDIA6 and spastin coordinate to drive both neurite branch formation and outgrowth.

The damage associated with SCI can be extremely severe, resulting in substantial functional impairment including permanent motor dysfunction in affected patients owing to the limited regenerative responses engaged in humans following such injury (Zhu et al., 2020; Bisicchia et al., 2022). The pathogenesis of SCI is linked to mechanisms that govern both primary and secondary injury (Walsh et al., 2021; Ding and Chen, 2022), with secondary damage in particular contributing to more widespread neuronal death, expanding the affected region of the spinal cord (Lai et al., 2021). These deleterious conditions can contribute to the buildup of unfolded or misfolded proteins, interfering with normal ER function and thereby activating ER-associated cell death (Oakes and Papa, 2015; Han et al., 2021; Mao et al., 2022). According to an increasing number of studies, the inhibition of ER stress-induced cell death can improve functional recovery following SCI (Wu et al., 2020; Saraswat Ohri et al., 2021). The protein disulfide isomerase family protein PDIA6 is an oxidoreductase capable of catalyzing disulfide bond formation and acting as a chaperone to protect against excessive unfolded protein aggregation (Okumura et al., 2015). The function of PDIA6 in SCI, however, is not clarified previously. In this current research, but LC-MS and bioinformatics analyses suggested that PDIA6 was upregulated at the mRNA and protein levels in damaged spinal cord tissues in a rat model of SCI, suggesting a potential role for PDIA6 as a regulator of SCI pathogenesis.

In previous studies, we found that spastin serves as an important regulator of SCI through its ability to regulate microtubule dynamics and to thereby promote the formation and outgrowth of neurites (Ji Z. S. et al., 2021). We therefore hypothesized that PDIA6 may be capable of interacting with spastin to shape the functional recovery of injured neurons. Through a pull-down assay conducted using GST-spastin and rat spinal cord lysates, LC-MS revealed PDIA6 to precipitate with spastin, consistent with our hypothesis. Subsequent co-IP and GST pull-down assays further confirmed these interactions between spastin and PDIA6, and these two proteins were found to co-localize with one another in neurons.

As a protein that severs microtubules, spastin can disassemble long microtubules into shorter fragments that serve as nucleation templates to facilitate further microtubule growth (Roll-Mecak and Vale, 2006; Kapitein and Hoogenraad, 2015; Solowska and Baas, 2015). Several reports have demonstrated that a range of proteins can interact with spastin to modulate its ability to sever microtubules, thereby influencing neurite outgrowth from injured neurons (Ji et al., 2018; Ji Z. S. et al., 2021; Lawrence et al., 2021). As such, the ability of PDIA6 to regulate injured neuronal repair through interactions with spastin and associated regulation of microtubule severing was assessed by overexpressing spastin in COS-7 cells. This resulted in the disruption of the normal microtubular network within these cells consistent with the near-complete severing of longer microtubules. When PDIA6 was simultaneously knocked down, however, this spastin-mediated microtubule severing activity was markedly blunted, confirming the ability of PDIA6 to alter the ability of spastin to sever these microtubules. As such, these data support a model wherein PDIA6 and spastin interact to control intracellular microtubule dynamics.

Promoting axonal regeneration to restore the integrity of neural networks is thought to represent a promising approach to achieving functional recovery following SCI (Li et al., 2017; Sekine et al., 2022). Our previous studies have shown that synergistic interactions between proteins were capable of promoting axonal branching and outgrowth in hippocampal neurons damaged following glutamate exposure (Wu et al., 2021; Ji et al., 2022). Here, the overexpression of PDIA6 in neurons was sufficient to promote axonal outgrowth in damaged neurons, while this beneficial effect was reversed when spastin was silenced in these same cells. Similarly, PDIA6 knockdown impaired neurite outgrowth of damaged neurons. Together, these data thus suggest that PDIA6 promotes neuronal outgrowth through a mechanism dependent on its ability to interact with spastin.

In conclusion, these data suggest that both PDIA6 and spastin are important regulators of neuronal responses to SCI, with the interaction between these two proteins serving to regulate intracellular microtubule dynamics, thereby controlling neurite outgrowth and thus potentially influencing SCI-related repair responses. PDIA6 can additionally promote injured neuron recovery through the regulation of spastin functionality *in vitro*, although this functional activity remains to be confirmed *in vivo.* Future research focused on the molecular mechanisms underlying the observed interactions between PDIA6 and spastin thus has the potential to guide the design of novel therapeutic drugs and other interventions aimed at improving neuronal regeneration following SCI.

## Data availability statement

The datasets presented in this study can be found in online repositories. The name of the repository and accession number can be found below: ProteomeXchange, http://www.proteomexchange.org/, PXD035706.

## Ethics statement

The studies involving animals were reviewed and approved by the Ethics Committee of Jinan University.

## Author contributions

ZJ, HL, and GZ: study conception and design. JL, CP, and MX: experiment implementation. JL, KW, GL, and HY: data acquisition. TC, QL, and JL: data analysis. JL and ZJ: manuscript writing. All authors reviewed and approved the final version of the manuscript.
